# Semi-automated fact-checking of nucleotide sequence reagents in biomedical research publications: The Seek & Blastn tool

**DOI:** 10.1371/journal.pone.0213266

**Published:** 2019-03-01

**Authors:** Cyril Labbé, Natalie Grima, Thierry Gautier, Bertrand Favier, Jennifer A. Byrne

**Affiliations:** 1 Univ. Grenoble Alpes, CNRS, Grenoble INP, LIG, Grenoble, France; 2 Molecular Oncology Laboratory, Children’s Cancer Research Unit, Kids Research, The Children’s Hospital at Westmead, Westmead, New South Wales, Australia; 3 INSERM U1209/ CNRS UMR 5309, Univ. Grenoble Alpes, Grenoble, France; 4 Univ. Grenoble Alpes, Team GREPI, Etablissement Français du Sang, La Tronche, France; 5 Discipline of Child and Adolescent Health, Faculty of Medicine and Health, The University of Sydney, Westmead, New South Wales, Australia; Fred Hutchinson Cancer Research Center, UNITED STATES

## Abstract

Nucleotide sequence reagents are verifiable experimental reagents in biomedical publications, because their sequence identities can be independently verified and compared with associated text descriptors. We have previously reported that incorrectly identified nucleotide sequence reagents are characteristic of highly similar human gene knockdown studies, some of which have been retracted from the literature on account of possible research fraud. Because of the throughput limitations of manual verification of nucleotide sequences, we developed a semi-automated fact checking tool, Seek & Blastn, to verify the targeting or non-targeting status of published nucleotide sequence reagents. From previously described and unknown corpora of 48 and 155 publications, respectively, Seek & Blastn correctly extracted 304/342 (88.9%) and 1066/1522 (70.0%) nucleotide sequences and a predicted targeting/ non-targeting status. Seek & Blastn correctly predicted the targeting/ non-targeting status of 293/304 (96.4%) and 988/1066 (92.7%) of the correctly extracted nucleotide sequences. A total of 38/39 (97.4%) or 31/79 (39.2%) Seek & Blastn predictions of incorrect nucleotide sequence reagent use were correct in the two literature corpora. Combined Seek & Blastn and manual analyses identified a list of 91 misidentified nucleotide sequence reagents, which could be built upon through future studies. In summary, incorrect nucleotide sequence reagents represent an under-recognized source of error within the biomedical literature, and fact checking tools such as Seek & Blastn may help to identify papers and manuscripts affected by these errors.

## Introduction

As biomedical science increases in both volume and complexity, the problem of irreproducible and incorrect published results is also growing [1, 2]. Up to 50% of published pre-clinical research results have been estimated to be incorrect, leading to the possible waste of billion dollars of research funds per year [3, 4]. As the post-publication correction of errors remains highly problematic [1, 2], there is an urgent need to reduce and deter the publication of incorrect research findings.

While most incorrect research results likely arise through honest error, some incorrect results arise through different forms of research fraud [5, 6]. As a covert activity, research fraud is difficult to study, and therefore likely to be both under-reported and incompletely described [2, 7]. Approaches are being developed and applied to detect particular forms of research fraud such as plagiarism and intertextuality [8, 9] and image duplication [10]. However, additional tools are needed to detect other genuine errors or fraudulent practices, to both better estimate the true prevalence of research quality and deter specific practices in the future.

The problem of incorrect published research results is leading to the development of fact checking systems for research publications [11–13]. Elements to be submitted to fact checking should represent verifiable facts that are both important to broad target audiences, and likely to be incorrect sufficiently often to justify the process of fact checking. To date, research fact checkers have evaluated chemical data [11] or statistical analyses [12, 13], and the application of statistical fact-checkers has identified widespread incorrect reporting of statistical results [12, 13].

In the field of biomedical research, the majority of incorrect results are estimated to derive from the incorrect use of material standards and experimental reagents [2, 3]. The repeated use of incorrect or incorrectly described reagents is of particular concern, as this can produce reproducible yet incorrect results that can increasingly derail research progress over time [14]. The use of reagent identifiers to improve reporting transparency is an important step to improve research reliability and reproducibility [15]. However, many reagent identifiers cannot be submitted to fact checking, as their identities cannot be reliably substantiated from other independent information supplied in the publication. To our best knowledge, there are currently no automated or semi-automated fact-checking systems for any class of experimental reagent commonly described in biomedical publications.

We have recently recognized that published nucleotide sequence reagents fulfill the requirements of suitable templates for fact checking [16]. Nucleotide sequence reagents are short DNA or RNA sequences that are required for widely-used laboratory techniques such as gene knockdown and polymerase chain reaction (PCR) approaches. Gene knockdown and PCR techniques rely upon the correct design and experimental use of RNA targeting reagents and PCR primers, respectively [17–19]. As the nucleotide sequences of these reagents define their identity and possible experimental use, each published reagent descriptor is recommended to be accompanied by its corresponding nucleotide sequence [18]. This pairing of reagent descriptors and nucleotide sequences allows the identities of published nucleotide sequence reagents to be independently verified.

Published PCR primer and RNA targeting sequences may also be incorrect sufficiently often to warrant fact-checking. Most nucleotide sequences cannot be easily read or understood by eye, because of codon redundancy, multiple possible reading frames, and other factors. This lack of visually apparent sense could allow incorrect nucleotide sequences to go unnoticed in manuscripts, and subsequently in publications. Different types of errors can also affect nucleotide sequences, which could further increase the prevalence of incorrect reagents within the literature. The equivalent of spelling mistakes (nucleotide substitutions, deletions or insertions) can be accidentally introduced into nucleotide sequences [17, 20], errors that we will describe as “typographic”. Nucleotide sequences can also be wrongly identified [16, 17]. For example, a reagent sequence may correspond to a different gene from that claimed, or a supposedly non-targeting control reagent may show significant homology to a known gene [16]. “Typographic” errors typically produce less efficient reagents, by reducing the reagent’s ability to bind its genetic target [17, 20, 21], whereas wrongly identified reagents could bind unexpected targets and produce irrelevant results [16]. In summary, because of the very frequent application of techniques that rely upon nucleotide sequence reagents, combined with the different types of visually hidden errors that can affect these reagents, the prevalence of incorrect nucleotide sequence reagents within the literature could be under-estimated. This is also suggested by the small number of studies that have focused on this problem [16, 20].

We have previously reported that incorrectly identified nucleotide sequence reagents were a frequent characteristic of a cohort of 48 highly similar human gene knockdown studies [16]. Incorrectly identified targeting and non-targeting sequences for gene knockdown experiments and targeting RT-PCR primers were found by manually comparing their stated identities with their verified identities according to blastn analyses [16]. Mismatches between blastn-confirmed sequence identities and their reported identities and experimental use rendered particular experimental results impossible, such as obtaining different results when the same shRNA was employed as both a targeting shRNA and a non-targeting control [16, 22]. The similarities between these 48 publications, coupled with frequent nucleotide sequence reagent errors, led to the hypothesis that these experiments may not have been performed as described [16]. As a result of subsequent communications with journal editors, 17 publications [22–38] have been retracted, including 14 of the 48 publications originally described [22–35]. In addition, 5 expressions of concern have been published [39–43] and 4 publications have been corrected [44–47]. Another similar publication was recently retracted by the authors due to the use of an incorrectly identified nucleotide sequence reagent [48].

As most (38/48, 79%) of the highly similar papers described by Byrne and Labbé [16] incorrectly identified one or more nucleotide sequence reagents, screening publications for incorrect nucleotide sequences may be a useful strategy to identify incorrect or potentially fraudulent papers. We recognized that our initial report was limited in scope, through the use of manual analysis [16]. A semi-automated approach to detect incorrect nucleotide sequence reagents would present advantages of increased throughput, and the capacity for knowledge discovery. We therefore describe a tool, Seek & Blastn (S&B), to facilitate the identification of publications where the claimed status of a nucleotide sequence does not match its verified status according to blastn analysis. This report describes the development of S&B, its use to screen two literature corpora, the current strengths and weaknesses of the tool, and how S&B may be applied to improve the description of nucleotide sequence reagents within the literature.

## Results

### Description of Seek & Blastn outputs

**Fig 1:**
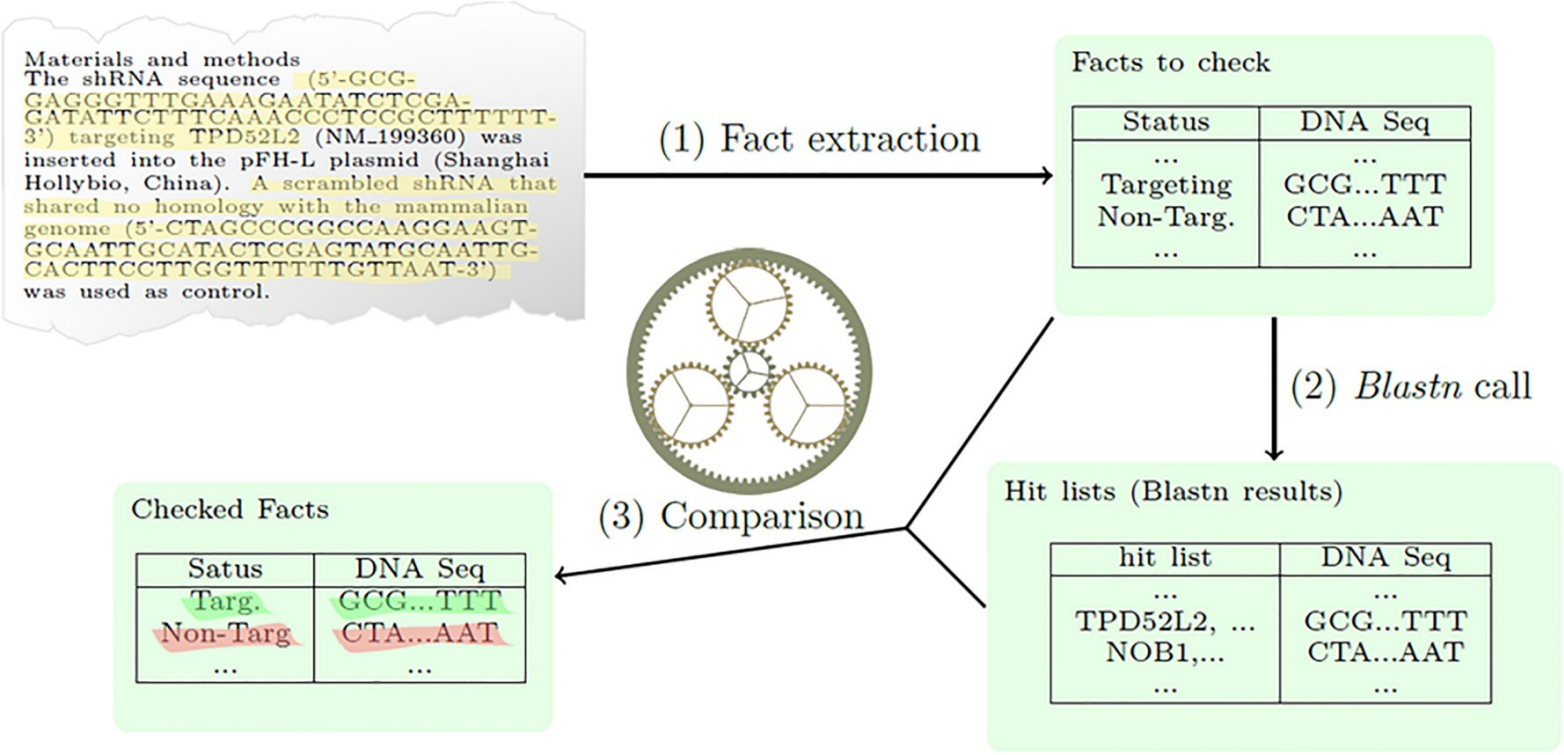
Diagrammatic illustration of the key steps of Seek & Blastn (S&B). S&B extracts facts to check from published text (nucleotide sequences with associated targeting/ non-targeting status), and then performs blastn analyses and fact checking.

We built the S&B tool to identify incorrect nucleotide sequence reagents in sets of publications in pdf format. The S&B tool involves 3 steps: identification and extraction of nucleotide sequences from text together with the associated claimed status of targeting or non-targeting (T/NT), blastn analyses [49], and then fact-checking to confirm or deny the usage claim associated with each extracted nucleotide sequence (Fig 1). The tool is freely accessible through the S&B website [50].

**Fig 2:**
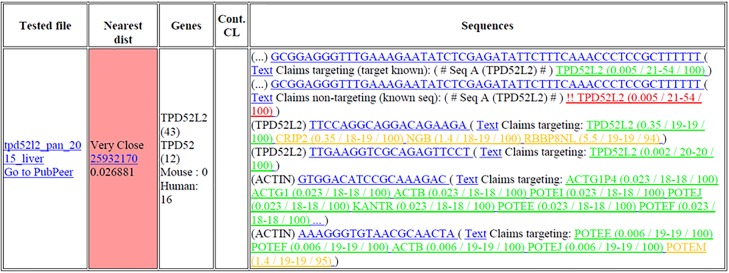
Example of Seek & Blastn (S&B) output for a retracted Corpus P paper (Ref. [22]). Columns shown from left to right are: “Tested file”; “Nearest dist”, which provides intertextual distance analysis results [9]; “Genes”, which provides gene and species identifiers extracted from the text; “Cont. CL”, which provides identifiers that correspond to contaminated or misidentified cell lines; and “Sequences”, which lists all nucleotide sequences that were extracted, and their corresponding blastn results. The tested publication forms part of the reference cohort [16], and its closest match is the same publication within the reference cohort. The tested pdf included 6 nucleotide sequences, which were correctly extracted and identified by S&B. Two extracted sequences were recognized as a previously reported sequence (SeqA) [16], and a mismatch was detected between the claimed non-targeting status and the blastn identity, as shown in red hypertext.

After uploading pdf files to the S&B website [50], S&B outputs are provided in tabular form (Fig 2, S1 and S2 Tables). Outputs for each paper are shown in a discrete row divided into 5 columns (Fig 2). The first output column “Tested file” provides links to (i) the pdf file uploaded, and (ii) the PubPeer website [51] (Fig 2), although no PubPeer notifications have been made directly from S&B by the authors to date. The second column “Nearest dist” shows the results of intertextual distance analysis [9], to describe the degree of textual similarity between the query publication and the most similar publication in the previously described reference cohort [16]. The PubMed ID of the most similar reference cohort publication is provided, and intertextual distance analysis results are shown as a numerical value between 0 and 1 (Fig 2), where smaller intertextual distance values indicate a greater degree of text similarity [9]. The similarity class is described as “ok” for intertextual distances > 0.5, “close” for intertextual distances from 0.44–0.50 and “very close” for intertextual distances <0.44, based on the distribution of intertextual distance scores for a consecutive series of 4094 publications in the International Journal of Clinical and Experimental Medicine [16]. The third column “Genes” lists individual gene identifiers extracted from the text, and terms “human” and/or “mouse”, with the number of instances shown next to each identifier (Fig 2). The 4th column “Cont. CL” lists any identifiers that are consistent with either contaminated or misidentified cell lines [52]. If no such identifiers are found, this column is left blank (Fig 2). Blastn results are listed in the 5th “Sequences” column (Fig 2). As most publications that include nucleotide sequences will describe more than one sequence (due to, for example, PCR primers being used in pairs), the S&B results for each sequence extracted are listed in rows within this column (Fig 2).

Within the “Sequences” column, the gene symbol extracted from the text that was found nearest each individual sequence is shown in brackets to the left of the sequence (Fig 2). Where no gene identifier was extracted, this is indicated by empty brackets (Fig 2). The extracted sequence is then shown in blue hypertext. Where it is recognized that the sequence has been incompletely or incorrectly extracted, through either being <14 nucleotides, or >91 nucleotides, the sequence is followed by the text “(Seq. not correctly extracted (Char/long/short)”. Other nucleotide sequences have a number of additional outputs. Firstly, the sequence links to the Google Scholar output for this sequence when used as a search query (Fig 2). The output also provides a hyperlink to the query text, and the detected corresponding text claim (“Claims targeting”, “Claims non-targeting”, or “No claim detected”). This is followed by the gene name corresponding to the first significant blastn hit, and then in brackets (i) the smallest associated e-value, (ii) the number of sequential nucleotides within the query sequence mapping to the blastn hit, (iii) the length of the query sequence, and (iv) the percentage sequence identity (Fig 2). Each gene name is hyperlinked to the associated blastn result describing all gene hits (Fig 2). This feature supports the manual confirmation of blastn results, and the identification of targeting sequences that may target a gene other than that described in the text.

Colour-coded hypertext within S&B outputs denotes the predicted relationship between an extracted claim and the blastn results (Fig 2). Where no claimed T/NT status is extracted for an individual sequence, indicated by “Undetected claim”, blastn results are shown in grey hypertext. Where a claimed T/NT status is provided, either as “Claims targeting” or “Claims non-targeting” (Fig 2), blastn results are shown in green, orange or red hypertext. Green hypertext shows blastn results that support the claimed T/NT status, written as either “Claims targeting”, “Claims non-targeting”, “No clear target”, or “No hits found” (Fig 2). Orange hypertext denotes lower-significance blastn hits. Red hypertext shows blastn results that conflict with the claimed T/NT status. Claimed targeting sequences lacking clear targets are indicated by “!! No clear target” or “!! No hits found” in red hypertext (Fig 2). More detailed explanations are provided in the Materials and Methods section below.

### Text corpora

S&B was tested using two text corpora, Corpus P (Problematic) and Corpus U (Unknown) (Table 1, S3 Table). Corpus P represents the 48 publications reported by Byrne and Labbé [16], of which 38 papers include incorrectly described nucleotide sequences. Corpus P publications commonly describe gene knockdown experiments employing gene silencing and non-targeting shRNA or siRNA (Table 1) [16]. These studies also performed RT-PCR analyses to confirm the degree of gene silencing, which were compared with control RT-PCR experiments examining ubiquitously expressed “housekeeping” genes [16]. As a defined corpus with a high incidence of incorrect sequence use, Corpus P was used to incrementally improve S&B, and the described S&B version was then applied to both Corpus P and Corpus U (Table 1). Corpus U was retrieved using papers from Corpus P and the “PubMed similar” functionality, together with Google Scholar queries of misidentified sequences. Any papers that were either common to Corpus P [16] or that had been previously subjected to manual analysis were excluded. Corpus U included a broader range of publications than Corpus P, as reflected by a broader range of publication dates, a larger number of individual journals, and other factors (Table 1).

**Table 1:** Descriptions of Corpus P and Corpus U analysed by Seek & Blastn.

Corpus features	Corpus P	Corpus U
Number of publications	48	198
Number of journals	25	90
Publication dates (years)	2012–2017	2001–2016
Median (range) Journal Impact Factor	1.929 (0.833–3.650)	3.300 (0.833–41.577)
Number (%) publications relevant to human cancer	48/48 (100%)	174/198 (87.9%)
Number (%) publications that include nucleotide sequences	48/48 (100%)	155/198 (78.3%)
Number (%) publications that employ siRNA/ shRNA/ PCR^a^	48/48 (100%)	175/198 (88.4%)

^a^PCR refers to the techniques of PCR, RT-PCR, qPCR and methylation-specific PCR

### Nucleotide sequence and targeting/ non-targeting status extraction by Seek & Blastn

Manual analyses indicated that Corpus P and Corpus U included 342 and 1522 nucleotide sequences, respectively (Table 2), which were distributed across 48 (100%) and 155 (78.3%) papers in Corpus P or Corpus U (Table 1). As 155/198 Corpus U publications included nucleotide sequences, we will henceforth refer to Corpus U as containing 155 papers.

**Table 2:** Seek & Blastn nucleotide sequence and associated status extraction (targeting versus non-targeting) from Corpus P and Corpus U publications.

Seek & Blastn sequence and status extraction	Corpus P (n = 48 papers) (n = 342 sequences)^a^	Corpus U (n = 155 papers) (n = 1522 sequences)^a^
**No error in sequence and status extraction**	**304/342 (88.9%)**	**1066/1522 (70.0%)**
**Error in sequence and/or status extraction**^b^	**38/342 (11.1%)**	**456/1522 (30.0%)**
Targeting/non-targeting claim not detected	21/342 (6.1%)	224/1522 (14.7%)
Missed sequences	11/342 (3.2%)	146/1522 (9.6%)
Sequences incorrectly extracted	10/342 (2.9%)	73/1522 (4.8%)
Sequence split	6/10 (60.0%)	28/73 (38.4%)
Loss of nucleotides^c^	4/10 (40.0%)	12/73 (16.4%)
Addition of nucleotides	0/10 (0%)	41/73 (56.2%)
Targeting/non-targeting claim incorrectly assigned	1/342 (0.3%)	14/1522 (0.9%)
**Error in gene identification**	**87/342 (25.4%)**	**865/1522 (56.8%)**
Gene identifier incorrectly assigned^d^	22/342 (6.4%)	83/1522 (5.5%)
Gene identifier not detected for targeting sequence	65/294^e^ (22.1%)	782/1452^e^ (53.9%)

^a^Refers to the total number of sequences present in each corpus

^b^More than one type of error in sequence or status extraction occurred for some sequences

^c^Includes ‘d’ of dTdT from sequences described in Corpus U only

^d^Includes assignment of the incorrect gene identifier to a targeting sequence and assignment of a gene identifier to a non-targeting sequence

^e^Number of targeting sequences in Corpus P or Corpus U

S&B correctly extracted 88.9% (304/342) or 70.0% (1066/1522) of the nucleotide sequences and their associated T/NT status from Corpus P or Corpus U, respectively (Table 2). For the remaining nucleotide sequences, errors were made in sequence extraction and/or recognition of the associated T/NT status (Table 2). For both corpora, the most frequent error was the failure to detect an associated T/NT claim within the text, followed by missed sequences, followed by sequences that were incorrectly or incompletely extracted (Table 2). In the case of Corpus P, most incorrectly or incompletely extracted sequences were split into at least 2 smaller sequences, whereas most incorrectly or incompletely extracted Corpus U sequences showed the addition of one or more nucleotide residues (Table 2).

S&B also extracts and reports gene identifiers within the text, and associates these with predicted targeting sequences (Fig 2). Whereas most Corpus P targeting sequences were associated with a gene identifier, more than half of the Corpus U targeting sequences were not associated with a gene identifier (Table 2).

### Manual verification of Seek & Blastn results-individual nucleotide sequences

**Fig 3:**
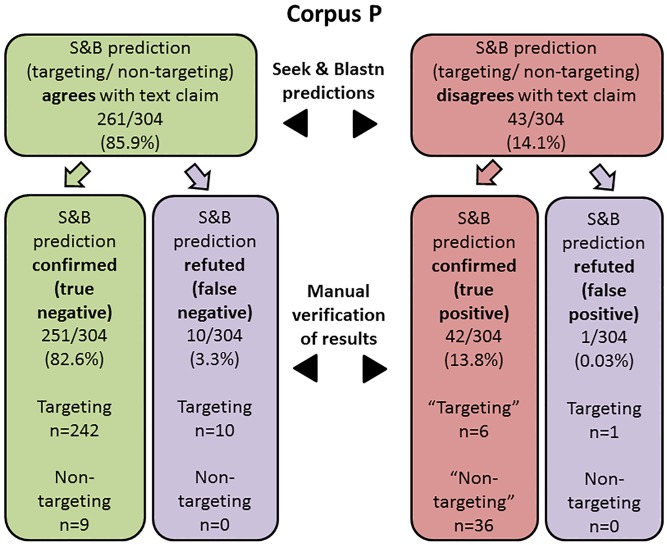
The proportions of Seek & Blastn (S&B) status predictions for 304 correctly extracted Corpus P sequences that were either confirmed or refuted by manual analyses. Predictions were classified as either true negative, false negative, true positive or false positive outcomes. The numbers of targeting and non-targeting sequences for each of the 4 possible outcomes are listed separately. Where sequences were correctly flagged by S&B as true positives, “Targeting” and “Non-targeting” refer to the incorrect claimed status in the relevant publication.

**Fig 4:**
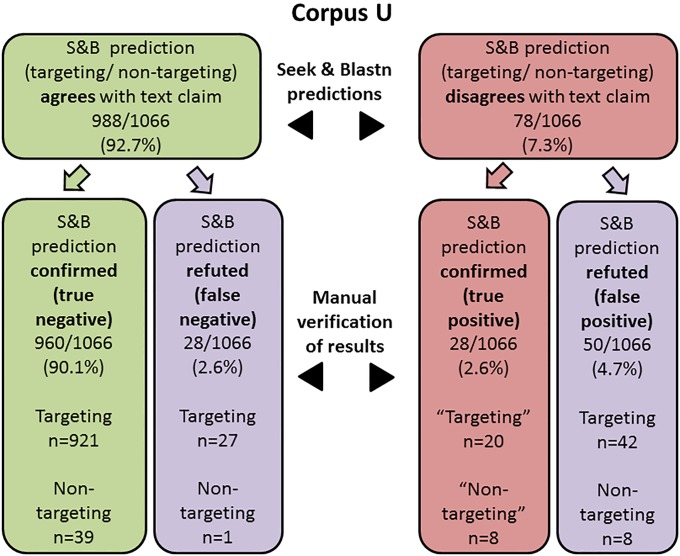
The proportions of Seek & Blastn (S&B) status predictions for 1066 correctly extracted Corpus U sequences that were either confirmed or refuted by manual analyses. Predictions were classified as either true negative, false negative, true positive or false positive outcomes. The numbers of targeting and non-targeting sequences for each of the 4 possible outcomes are listed separately. Where sequences were correctly flagged by S&B as true positives, “Targeting” and “Non-targeting” refer to the incorrect claimed status in the relevant publication.

Manual sequence extraction and independent blastn analyses were performed to cross-check the T/NT status predictions made by S&B, at the level of both nucleotide sequences (Figs 3 and 4) and publications (see below). In the case of nucleotide sequences, these analyses considered those sequences that manual analyses confirmed to have been correctly extracted by S&B, and also associated with a predicted T/NT status (Figs 3 and 4).

Most correctly extracted sequences from Corpus P (Fig 3) and Corpus U (Fig 4) were flagged by S&B as having blastn-confirmed identities that were concordant with the text T/NT claim. Almost all S&B predictions of concordance applied to claimed targeting sequences in both Corpus P and Corpus U, and almost all of these predictions were confirmed through manual analyses (Figs 3 and 4). Much smaller proportions of Corpus P and Corpus U sequences with concordant T/NT claims were predicted to be non-targeting sequences, and most or all of these predicted non-targeting sequences were correctly identified by S&B (Figs 3 and 4). Most false negative S&B decisions arose because claimed targeting sequences showed homology to genes other than those described in the text (Figs 3 and 4, S1 Table). These decisions were categorized as false negative decisions (Figs 3 and 4), even though S&B cannot automatically flag targeting sequences that target different genes from those claimed in the text.

The remaining minority of extracted Corpus P and Corpus U sequences were flagged as having a T/NT status that conflicted with the claimed status in the text (Figs 3 and 4). In Corpus P, 36/43 of these sequences represented “non-targeting” sh/siRNA sequences that blastn analyses indicated to target a human gene, all of which were correctly flagged by S&B (Fig 3). The remaining 7 Corpus P sequences were “targeting” sequences for which targets were not identified by S&B, and 6/7 of these “targeting” sequences were correctly flagged by S&B (Fig 3). In contrast to Corpus P, most Corpus U sequences with predicted conflicting status were claimed targeting sequences (Fig 4). While around one third of these claimed targeting sequences were correctly flagged, the remaining sequences were incorrectly flagged by S&B (Fig 4), frequently because sequences were of non-human origin (Table 3). Of the 16 claimed non-targeting Corpus U sequences, 8 sequences each were flagged correctly or incorrectly (Fig 4). Overall, the precision of S&B predictions was 96.4% (293 correct predictions/ 304 predictions) for Corpus P, and 92.7% (988 correct predictions/ 1066 predictions) for Corpus U (Figs 3 and 4).

**Table 3:** Numbers and proportions of Corpus P and Corpus U papers that were correctly or incorrectly flagged by Seek & Blastn (S&B).

Papers flagged for incorrect nucleotide sequence use	Corpus P (n = 48 papers)	Corpus U (n = 155 papers)
**Papers flagged by S&B and manual analyses (true positives)**	**38/48 (79.2%)**	**31/155 (20.0%)**
**Papers flagged by S&B analysis only (false positives)**	**1/48 (2.1%)**	**40/155 (25.8%)**
Gene identifier incorrectly extracted by S&B	1/1 (100%)	14/40 (35.0%)
Targeting/non-targeting claim incorrectly assigned by S&B	0/1 (0%)	11/40 (27.5%)
Non-human genome	0/1 (0%)	6/40 (15.0%)
Nucleotide mismatch prevented blastn identification	0/1 (0%)	5/40 (12.5%)
Experimental purpose incompatible with blastn identification	0/1 (0%)	5/40 (12.5%)
Mutagenesis primer	0/1 (0%)	1/40 (2.5%)
Methylation-specific PCR primer	0/1 (0%)	1/40 (2.5%)
Other ^a^	0/1 (0%)	3/40 (7.5%)
Sequence incorrectly extracted by S&B	0/1 (0%)	2/40 (5.0%)
Vector sequence	0/1 (0%)	2/40 (5.0%)
Genomic DNA target not recognised	0/1 (0%)	2/40 (5.0%)
**Papers flagged by manual analysis only (false negatives)**	**0/48 (0%)**	**8/155 (5.2%)**
Sequence incorrectly extracted by S&B	0/48 (0%)	4/8 (50.0%)
Missed sequence by S&B	0/48 (0%)	3/8 (37.5%)
Incorrect gene decision in absence of gene identifier	0/48 (0%)	1/8 (12.5%)
**Papers not flagged by S&B and manual analyses (true negatives)**	**9/48 (18.8%)**	**76/155 (49.0%)**

^a^ Includes intron-exon boundary sequence, binding consensus sequence, CpG oligodeoxynucleotide (ODN)

### Incorrectly identified nucleotide sequence reagents in Corpus P and Corpus U

Through S&B and manual analyses, we derived a list of 91 incorrectly identified nucleotide sequence reagents (S4 Table). Sequences were considered to have been identified by S&B (n = 56 sequences) if they were flagged as having discordant T/NT status, or if the gene identifier assigned to the sequence by S&B was not present in the provided blastn output. Sequences were considered to have been manually identified (n = 36 sequences) if the sequence was not extracted, or was incorrectly extracted and/or extracted with an undetected claim by S&B, and/or was not associated with any gene identifier by S&B and was not otherwise flagged. One RT-PCR primer was independently identified in two different publications using S&B or manual analysis (S4 Table).

Of these 91 independent reagents, 26 (28.6%) represented incorrectly identified sh/siRNA targeting reagents and the remaining 65 (71.4%) sequences were incorrectly identified PCR primers, including one mutagenesis primer. All PCR primer sequences were cross-checked against the PrimerBank database [53], and a partial overlap was identified for one PCR primer only (S4 Table). The described T/NT status of 48/91 (52.7%) sequences was found to be incorrect, either as “non-targeting” sh/siRNA sequences that were identified to be targeting reagents, or as “targeting” reagents (sh/siRNA sequences, PCR and mutagenesis primers), for which no target for the claimed species could be identified. The remaining 43/91 (47.3%) sequences were indicated to target a gene or sequence other than that described within the text. A significantly higher proportion of sequences with incorrect T/NT status was identified by S&B (35/56, 62.5%), whereas most incorrectly-identified sequences found only through manual analyses (23/36, 63.9%) targeted a gene or sequence other than that described in the text (Fisher’s Exact test, p = 0.0186, n = 91). This result supports both the fact that S&B was written to identify sequences with incorrect T/NT status, and that manual analyses are required to identify targeting sequences that show homology to a different gene or sequence from that described.

### Manual verification of Seek & Blastn results- flagged publications

We then compared the proportions of Corpus P and Corpus U papers that were correctly flagged by S&B as describing one or more incorrect nucleotide sequence reagents, compared with the proportions of papers that were flagged by manual analysis. A paper was considered to have been correctly flagged if it contained at least one incorrect nucleotide sequence that was correctly flagged by S&B, regardless of whether the paper also contained any sequence(s) that had been incorrectly flagged. A paper was considered to have been incorrectly flagged by S&B if the paper contained one or more incorrectly flagged sequences, and no correctly flagged sequences.

All 38 Corpus P papers that contained at least one incorrect nucleotide sequence claim were correctly flagged by S&B (Table 3). S&B incorrectly flagged one additional paper, which corresponded to a precision rate of 97.4% (38 correct/ 39 predictions). Manual analyses flagged 39/155 (25.2%) Corpus U papers, and 31/39 (79.5%) of these Corpus U papers were correctly identified by S&B (Table 3). However, more incorrectly flagged than correctly flagged Corpus U papers were identified by manually checking S&B results, corresponding to a precision of 39.2% (31 correct/ 79 predictions) (Table 3). Detailed analysis of S&B outputs indicated several explanations for these false positive results, including the incorrect assignment of targeting versus non-targeting claims, and other errors (Table 3).

### Proportions of Corpus P and Corpus U papers with apparent typographic versus sequence identity errors

We compared the proportions of papers in Corpus P and Corpus U that described nucleotide sequence(s) with typographic versus identity errors. While we recognize that a more expert understanding of some nucleotide sequence reagents described in Corpus U papers could explain some apparent typographic errors [21], we considered such errors to include nucleotide substitutions (1–6 nucleotides/ sequence), deletions (1–3 nucleotides/ sequence), or additions to either 5’ or 3’ sequence ends (1–15 nucleotides/ sequence) that resulted in mismatches between nucleotide sequences and their predicted targets (Table 4).

**Table 4:** Corpus P and Corpus U papers with apparent nucleotide sequence typographic versus identity errors.

Class of nucleotide sequence error^a^	Corpus P (n = 48 papers) (n = 38 papers with nucleotide sequence error(s))^b^	Corpus U (n = 155 papers) (n = 39 papers with nucleotide sequence error(s))^b^	
	Papers with error	Papers with error	Intertextual distance <0.5
**Sequence typographic errors**	3	18	11/18 (61.1%)
Substitution of nucleotides	2/3 (66.7%)	8/18 (44.4%)	2/8 (25.0%)
External addition of nucleotides	1/3 (33.3%)	11/18 (61.1%)	4/11 (36.4%)
Deletion of nucleotides	0/3 (0%)	4/18 (22.2%)	2/4 (50.0%)
Internal addition of nucleotides	0/3 (0%)	1/18 (5.6%)	1/1 (100%)
**Sequence identity errors**	38	38	22/38 (57.9%)
Targeting sequence targets incorrect gene	6/38 (15.8%)	19/38 (50.0%)	8/19 (42.1%)
Gene not described in paper	6/6 (100%)	12/19 (63.2%)	5/12 (41.7%)
Gene described in paper	0/6 (0%)	7/19 (36.8%)	3/7 (42.9%)
“Non-targeting” sequence targets gene	37/38 (97.4%)	10/38 (26.3%)	10/10 (100%)
Gene not described in paper	34/37 (91.9%)	8/10 (80.0%)	8/8 (100%)
Gene described in paper	3/37 (8.1%)	2/10 (20.0%)	2/2 (100%)
“Targeting” sequence is non-targeting	1/38 (2.6%)	16/38 (42.1%)	9/16 (56.3%)

^a^Some papers included more than one class of nucleotide sequence error

^b^Numbers of papers with nucleotide sequence errors represent the combined results of S&B and manual analyses

All 38 Corpus P papers that described incorrect nucleotide sequence reagents included at least one incorrectly identified reagent (Table 4). In most cases, the genes that these reagents were predicted to target were not described in the corresponding papers (Table 4). Almost all Corpus P papers with incorrectly identified reagents described “non-targeting” sequences that blastn analyses predicted to target a human gene [16] (Table 4), with a smaller proportion describing wrongly identified targeting sequences (Table 4). A minority of Corpus P papers contained nucleotide sequences with apparent typographic errors, which were either nucleotide substitutions or additions (Table 4). In contrast, approximately half of the Corpus U papers with incorrect nucleotide sequence reagents described one or more reagents with apparent typographic errors, which took the form of nucleotide substitutions, external or internal nucleotide additions or deletions, or internal sequence duplications (Table 4). Most of these papers also described wrongly identified nucleotide sequences, which were either incorrectly identified targeting sequences, “targeting” sequences that were indicated to be non-targeting, or “non-targeting” sequences that were predicted to target a human gene (Table 4). In most cases, the genes predicted to be targeted by incorrect targeting or “non-targeting” reagents were not described in the corresponding papers (Table 4).

S&B measures the intertextual distance [9] between each analyzed paper and a reference group of single gene knockdown publications [16], and we had previously considered that papers with intertextual distances of <0.5 were highly similar to reference publications [16]. Intertextual distance analysis indicated that all Corpus P papers and approximately half of the Corpus U papers with nucleotide sequence identity errors were highly similar to reference publications (Table 4) [16]. Similarly, approximately half of Corpus U papers with either (i) apparent typographic sequence errors, (ii) incorrectly identified targeting sequences and/or (iii) “targeting” sequences that were predicted to be non-targeting were also highly similar to reference publications (Table 4). In contrast, all 10 Corpus U papers describing incorrect “non-targeting” reagents were highly similar to reference papers (Table 4). As such, a significantly greater proportion of Corpus U papers with incorrect “non-targeting” reagents were highly similar to reference publications (10/10 papers), compared with the proportion of Corpus U papers that described other wrongly identified nucleotide sequence reagents (12/28 papers) (Fisher’s exact test, p = 0.0019, n = 38).

## Discussion

We report the derivation and testing of the novel open-access S&B tool that permits the semi-automated fact checking of nucleotide sequence reagents, a class of experimental reagent that has been employed in hundreds of thousands of biomedical research publications. The undetected reporting of incorrect nucleotide sequence reagents could lead to such results misdirecting future research, and to the continued use of incorrect reagents in future studies. The S&B tool therefore directly addresses the larger problem of material reagents and standards representing the major source of incorrect published results from pre-clinical research [3, 4].

When considering our results, we must first highlight that S&B has been applied to only selected text corpora, and an overall small number of papers, on account of the laborious nature of manually cross-checking large numbers of diverse papers. Furthermore, because of the manner in which Corpus U papers were retrieved, the reported frequencies of nucleotide sequence reagent errors are unlikely to correspond to those in the total population of journal articles. We also recognize that S&B is yet to be applied to corpora of either consecutively published or randomly sampled publications, to describe baseline frequencies of incorrect nucleotide sequences, and how these frequencies may differ between fields or journals. Nonetheless, our preliminary results indicate that nucleotide sequence reagent errors may occur more frequently than expected in some publication types, and that the potential impact of these errors may be unappreciated.

### Seek & Blastn performance and comparisons with text mining

We considered the performance of S&B both in terms of its capacity to correctly extract and flag individual nucleotide sequences, and to correctly flag publications that included one or more incorrect nucleotide sequence reagents. S&B was optimized for the analysis of Corpus P, which consists of what we have previously described as single gene knockdown papers [16]. The S&B version that we have reported correctly extracted the majority (88.9%) of nucleotide sequences present in Corpus P, and their associated T/NT status. S&B also flagged all 38 papers that were flagged by manual analysis, and incorrectly flagged only one Corpus P paper, which represented a precision of 97.4%. While recognizing that S&B was developed using Corpus P as a test corpus, the automated analysis of single gene knockdown papers may be facilitated by their description of restricted numbers of nucleotide sequences, and their high degrees of textual similarity [16].

S&B was also applied to the larger, more diverse Corpus U, where S&B also correctly identified most (70.0%) nucleotide sequences and their associated T/NT status. However, the S&B error rate for sequence and/or T/NT status extraction for Corpus U (30.0%) was more than double that of Corpus P (11.1%), and all error types were more frequent in Corpus U than in Corpus P. These higher error rates, combined with particular issues only encountered for Corpus U papers such as the presence of non-human nucleotide sequences, were associated with a higher rate of falsely flagged Corpus U publications, and a reduced precision of 39.2%. Extending S&B blastn searches to include human genomic and non-human sequences may reduce the proportions of targeting sequences that are incorrectly flagged as non-targeting, which was a more frequent error for Corpus U than Corpus P.

The reduced precision achieved by S&B when applied to an unknown corpus reflects previous experiences from mining gene and protein symbols from text [54]. Over the past two decades, numerous tools have been described to extract and analyze gene and/or protein identifiers from publications for knowledge discovery [55–57], frequently using named entity recognition techniques to extract and classify designators from text [58]. Recognized challenges in the field of text mining gene or protein symbols that are also relevant to S&B include the incomplete uptake of standardized gene nomenclature within the literature [57, 59], the ambiguity of some gene identifiers [57, 60, 61], leading to gene symbols being incorrectly assigned non-gene meanings and vice versa [61], as well as heterogeneity of both document and data presentation [56]. Biocreative workshops have proposed challenges to overcome these problems [62], and have enabled the controlled comparison of different text mining systems that perform automated gene symbol recognition [54].

Regardless of the informatics approach taken, the process of text mining gene identifiers from the literature commonly assumes that reported gene identifiers are used correctly. However, wrongly identified nucleotide sequences [16] demonstrate that not all published information concerning gene identifiers and gene function is reliable. S&B therefore extends the reach of previously described text mining tools by adding nucleotide sequence fact checking capacity. Employing fact checking tools such as S&B prior to text mining may identify and then exclude unreliable publications, and thereby improve the validity of predictions made from text mining gene-associated information. Similarly, advances in text mining capacity could be incorporated into future versions of S&B, to improve the recognition and extraction of both gene symbols and associated experimental status claims.

### Nucleotide sequence errors in publications- consequences and underlying causes

This study of a relatively small number of papers supports the existence of undetected identity and typographic errors affecting published nucleotide sequence reagents. These different error types are likely be associated with different experimental consequences. While recognizing that some apparent typographic errors may be deliberately introduced into nucleotide sequence reagents in order to target particular gene transcripts or sequence variants, most typographic errors are likely to reduce reagent efficiency [21]. For example, this could occur if introduced sequence mismatches reduce the capacity of reagents to bind their intended targets. Such incorrect reagents may be detected by laboratory researchers in response to unexpectedly weak or negative experimental results, and these “loss of function” phenotypes could reduce the likelihood of such reagents being included in subsequent publications. In contrast, wrongly identified nucleotide sequence reagents may have more damaging consequences, by generating reproducible yet irrelevant and misleading results. Although unexpected results such as obviously incorrect PCR product sizes could flag the use of misidentified PCR primers, less obvious departures from anticipated experimental results could be overlooked. Furthermore, the effects of non-targeting sh/siRNA reagents are directly compared with those of targeting reagents [19], and as long as the “non-targeting” sequence does not also target the gene(s) under study, its false status is unlikely to be detected. Researchers may also be unlikely to verify the identities of non-targeting sequences, particularly those that have been repeatedly described within the literature, such as those predicted to target the *NOB1* or *TPD52L2* genes [16].

While the experimental consequences of identity versus typographic sequence errors are likely to differ, we predict that incorrect published nucleotide sequence reagents commonly reflect unintentional errors. We recognize that incorrect reagent descriptions could represent active attempts to sabotage the efforts of competitors [63, 64], but we could find no reports of errors being deliberately introduced into published nucleotide sequence reagents. It also seems unlikely that errors would be deliberately introduced into fraudulent manuscripts, as published errors can lead to fraud detection [65]. Instead, we hypothesize that wrongly identified nucleotide sequences in publications are more likely to reflect a lack of quality control and/or limited expert knowledge, which in the context of fraudulently produced research content could characterize both content producers and recipients. Similarly, typographic sequence errors seem likely to represent stochastic errors, possibly analogous to reported errors in published clinical trial identifiers [66]. Tracking shared errors or error types in larger paper cohorts may provide clues as to the origin of scientific content. For example, the text of all Corpus U papers that included incorrect “non-targeting” sh/siRNAs was highly similar to Corpus P reference publications [16], suggesting that the description of incorrect “non-targeting” reagents may be a hallmark of some publication types. However, the discovery of supposed “targeting” reagents that lacked identifiable targets in Corpus U papers, combined with the relative absence of such sequences from Corpus P papers, could indicate that other publication series exist within the literature.

### Future directions

The S&B tool is designed for use by any individual with a basic understanding of nucleotide sequence reagents and their experimental use, with such expertise being widely available within the biomedical research community. Functional genomics and other biomedical researchers may use S&B to check the validity of published reagents that are relevant to genes of interest, and/or genes with which they may be less familiar. As S&B performs reliably for the analysis of single gene knockdown papers, S&B could also be more broadly applied to identify possibly fraudulent publications within the literature. The capacity of S&B results to be extended lies in the Google Scholar search feature, which can identify other papers that have employed the same nucleotide sequence, and how this was employed [16]. Overall, we hope that the availability of S&B will lead researchers to more frequently check the identities of nucleotide sequence reagents, both when preparing manuscripts, and when planning experiments based upon published methods and results.

The application of S&B to two literature corpora identified 91 incorrectly identified nucleotide sequence reagents, supporting our hypothesis that the incorrect use of nucleotide reagents may be frequently undetected during peer review and post-publication. This reagent list can be used to establish an online, publicly available knowledgebase of wrongly identified nucleotide sequence reagents, to which researchers can refer when using S&B, or independently. Analogous to lists of misidentified or contaminated cell lines [52, 67], we anticipate that this list will serve as the basis for a resource that will grow over time, and increase awareness of the problem of incorrect nucleotide sequences within publications.

Incorrectly flagged papers in Corpus U highlight the need for more standardized descriptions of nucleotide sequences in publications, including standardized formatting and text descriptions of use. In contrast to some text mining tools that screen published abstracts [60], S&B must screen full text to extract nucleotide sequences for fact checking. Guidelines enforced by biomedical journals specifying the requirement for machine-readable, verifiable descriptions of nucleotide sequence reagents in publications could render nucleotide sequence reagents more transparent to automated analysis. Standardized reporting formats may also reduce human error by encouraging a greater degree of focus on the description of reagents, particularly those that are resistant to visual identification. Statements in manuscripts and/or letters to editors confirming that reported nucleotide sequence reagents have been verified by the author(s) will draw further attention to the possibility that nucleotide sequence reagents can be incorrectly reported. Guidelines for formatting and reporting nucleotide sequences would also remove the possibility that authors will omit nucleotide sequences from publications to avoid scrutiny. Finally, any guidelines developed for the nucleotide sequence formatting and reporting could also be relevant to the description of other verifiable reagents, as additional fact-checking tools are developed in future.

The availability of the first fact-checker for biomedical reagents, and moreover for a reagent class that is very widely used, is predicted to open a new field where fact checkers are developed to verify the identities of other experimental reagents. The core principles of S&B are fundamental components of fact checkers that could be developed for other verifiable reagents. We propose that nucleotide sequence reagents may be particularly prone to different classes of error, due to their lack of visually apparent sense. While the greater visual transparency of amino acid sequences may protect these sequences from errors, related fact checkers could be designed to determine whether peptide sequences are incorrectly described in publications. The comparison of error rates associated with the reporting of different reagent types could also help to design individual and/or shared solutions to these problems.

### Summary and conclusions

The S&B tool has the capacity to fundamentally alter knowledge of the extent of incorrect nucleotide sequence reagents within the literature, and of the possible extent of systematically fraudulent manuscript production. Our results suggest that visually hidden yet verifiable errors affecting nucleotide sequence reagents can be exploited to flag fraudulently produced papers. Tools such as S&B may prospectively deter publications that describe incorrect nucleotide sequence reagents, and may help to flag existing publications so that their conclusions can be re-evaluated. The identification of papers whose conclusions cannot be supported will prevent such papers from misdirecting future research efforts, and reducing the validity of predictions from text mining. Furthermore, as S&B combines the measurement of text similarity [9] and fact-checking of reagent identities, it can either be applied independently or used in parallel with other tools, such as those that detect duplicated images [10], and/or incorrect statistical results [12, 13]. In summary, the further development and broader application of S&B, along with fact checkers for other verifiable experimental reagents, is predicted to improve the reliability and integrity of published biomedical research, through an improved capacity to detect errors and research fraud.

## Materials and methods

S&B involves text extraction, text cleaning, sequence extraction, T/NT status identification, blastn results analysis and gene name extraction [50, 68, 69].

### Text preparation and processing

Raw text extraction from pdf’s using pdftotext [70] involves the loss of text indentations and table formatting, and the insertion of errors, such as header and footer lines within paragraphs. The resulting text is of poor quality for analysis and a cleaning step is required. Text cleaning removed lines that appeared several times in the document (such as journal headers and footers) and the references section, as this will not contain nucleotide sequences or their descriptions.

### Seek component: Sequence and associated targeting/ non-targeting claim extraction

**Fig 5:**
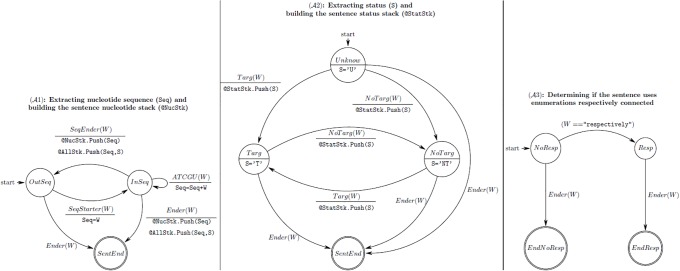
The three Seek & Blastn automata (A1, A2, A3) and associated stacks (StatStk, NucStack, AllStack). Circles represent states and arrows represent state transitions. The upper part of the label on an arrow specifies the property of the scanned word (W) that causes the transition. The lower part of the label described other actions triggered by the transition. The A1 automaton (shown at left) builds nucleotide sequence encounters in a sentence, extracting a nucleotide sequence (Seq) and building the sentence nucleotide stack (@NucStk). The A2 automaton (shown at centre) tracks the different targeting/ non-targeting status encounters when reading a sentence, which may be unknown (S = ʽU’), targeting (S = ʽT’) or non-targeting (S = ʽNT’). The automaton A3 (shown at right) tracks the use of the word “respectively” in the sentence. The ending state of this automaton is used to determine which stacks need to be used to decide the targeting/ non-targeting status of each nucleotide sequence.

A set of three automata were written to find nucleotide sequences in publications and to determine their claimed T/NT status. Claim extraction uses the three automata (A1, A2, A3) (Fig 5) together with three stacks (StatStk, NucStack, AllStack), which are used to store the sequences and the possible associated T/NT status. Each time a word (W) is read, this triggers a possible change of state for each automaton (Fig 5).

The A1 automaton builds nucleotide sequence encounters in a sentence. A nucleotide sequence usually starts with 5’ and ends with 3’ and may/ may not be split into “words”, through insertion of whitespaces after codon triplets, or at the end of a text line. The A2 automaton tracks the different T/NT status encounters when reading a sentence, which may be unknown (S = ʽU’), targeting (S = ʽT’) or non-targeting (S = ʽNT’) (Fig 5). A word (W) is considered a targeting (Targ(W) = true) or non-targeting marker (NoTarg(W) = true) if it is included within a predefined word set: “primer, siRNA, shRNA, targeting, silencing” for targeting; and “non-targeting, scramble(d), non-silencing” for non-targeting. The A1 automaton starts in the state OutSeq (outside a sequence) where the current sequence Seq has an undetermined value (Fig 5). When a word that starts a nucleotide sequence is encountered (e.g SeqStarter(W) is true if W starts with 5’, and/or has only ATCGU characters) the automaton is switched to the state InSeq (inside a sequence) and Seq is initialized with W. When in the InSeq state, when words containing only nucleotide symbols (ATCGU(W) is true) are encountered, S is updated. When W is the end of a sequence (SeqEnder(W) and Ender(W)) the current sequence Seq is stored in the NucStk and AllStk stacks together with the current status (Seq, S). AllStk is used (in A1) to store the current state of the status automaton (A2) each time a sequence end is encountered. The two other stacks (StatStk and NucStk) are used to keep the order in which values (claimed T/NT status and sequence) are encountered inside a sentence (Fig 5).

The automaton A3 tracks the use of the word “respectively” in the sentence (Fig 5). The ending state of this automaton is used to determine which stacks need to be used to decide the T/NT status of each nucleotide sequence. When the word “respectively” is used in a sentence describing the use of more than one nucleotide sequences, the two separated stacks are used. The first sequence encountered is associated with the first T/NT status encountered, the second sequence is associated with the second T/NT status, and so on. The final state of the A3 automaton is used to determine how to exploit stacks. If the word “respectively” has been used in the sentence, the two separated stacks (StatStk and NucStk) are used to determine the T/NT status of each sequence. Otherwise, each nucleotide sequence is associated with the T/NT status when the sequence was encountered in the sentence (using AllStk). When the sentence end is encountered (Ender(W)), all automata are switched to the terminal state.

### Blastn component

The blastn algorithm is widely used to verify whether a nucleotide sequence may target a particular gene or genomic sequence [49]. To determine if the extracted T/NT claim reflects the corresponding sequence’s verified identity, a blastn query is created for each extracted nucleotide sequence. Blastn queries analyze the human genomic and transcript database, as we have previously reported incorrect nucleotide sequences in human studies [16]. The criteria for a targeting sequence require either (i) 100% sequence identity over at least 15 consecutive nucleotides including the 3' end nucleotide of the extracted sequence, or (ii) two different sub-sequences of the sequence query matching a single target with inverted homology or (iii) 100% sequence identity over at least 17 consecutive nucleotides. Non-targeting sequences do not meet any of the above criteria. "No hit found" is called when blastn results indicated "no hit found", whereas "no clear target" is called when blastn results provide a non-significant hit, such as when sequence identity was <90% or was distributed across ≤14 consecutive nucleotides. Blastn results of lower significance are highlighted in orange hypertext, including sequences with ≥90% but <100% identity over at least 15 nucleotides at a distance of less than 3 nucleotides from the 3' end, or 100% identity over 16 consecutive nucleotides.

### Other Seek & Blastn outputs

We previously used Google Scholar to identify other instances of misidentified nucleotide sequence reagents within the literature, recognizing that Google Scholar did not identify all instances of these sequences, possibly because of formatting limitations [16]. For each sequence submitted to blastn analysis, a hyperlink is provided to the Google Scholar search results.

Gene names, contaminated cell lines and species are automatically extracted from the text using named entity recognition techniques [58, 71]. Named entity recognition was achieved using lists of known entities or *gazetteers*. The gazetteer for contaminated or misidentified cell line recognition was built on the Database of Cross-contaminated or Misidentified Cell Lines version 7.2 established by the International Cell Line Authentication Committee [52]. The gazetteer for gene symbol recognition was built on the approved symbol list established by the HUGO Gene Nomenclature Committee [72]. Because of word polysemy (for example, “WAS” is a gene name, “OF” is a HeLa-contaminated cell line), the surrounding words (using rule based entity extraction) were also used to reduce misinterpretation when identifying a proper entity.

### Manual verification of Seek & Blastn results

Publications were analysed manually to determine (i) rates of retrieval or recall for nucleotide sequence reagents (numbers of sequences retrieved by S&B divided by total number of sequences present in the text corpus, according to manual analyses), and (ii) precision rates for S&B predictions (number of correct S&B predictions divided by all S&B predictions). Errors made by S&B were identified as false positive, false negative and incorrect gene decisions. A false positive decision arose if S&B highlighted a sequence-T/NT status relationship as incorrect when this was actually correct. A false negative error arose if a sequence-T/NT status relationship was incorrect but flagged by S&B as correct. Incorrect gene errors occurred when blastn analyses predicted a sequence to target a gene or sequence other than that identified in the text. Although S&B was written to distinguish targeting from non-targeting sequences, incorrect gene errors were included within the reported false negative decisions.

### Statistical analyses

Fisher’s exact test was used to compare the proportions of (i) nucleotide sequences with incorrect T/NT status identified by S&B versus manual analyses, and (ii) Corpus U papers that were highly similar to reference publications [16], according to whether these papers described incorrect “non-targeting” reagents versus other wrongly identified nucleotide sequence reagents. As the compared samples were not randomly selected or independent, it should be noted that the corresponding confidence intervals may be biased upwards as a result.

## Supporting information

S1 Table Corpus P Seek & Blastn outputs. (XLSX)

S2 Table Corpus U Seek & Blastn outputs. (XLSX)

S3 Table List of PubMed ID’s corresponding to the 48 Corpus P and 155 Corpus U papers analyzed with Seek & Blastn. Please note that some publications listed were incorrectly flagged by Seek & Blastn, and others contain no nucleotide sequence reagent errors.
(DOCX)

S4 Table Incorrectly identified nucleotide sequence reagents from publications in Corpus P and Corpus U. (DOCX)
