# Supplementary material for: Semi-automated fact-checking of nucleotide sequence reagents in biomedical research publications: The Seek & Blastn tool
Source: PLoS One. 2019 Mar 1;14(3):e0213266. doi: 10.1371/journal.pone.0213266 (PMC6396917; doi:10.1371/journal.pone.0213266)
Supplement: S3 Table — Please note that some publications listed were incorrectly flagged by Seek & Blastn, and others contain no nucleotide sequence reagent errors. (DOCX) [file pone.0213266.s003.docx]

S3 Table**.** List of PubMed ID’s corresponding to the 48 Corpus P and 155 Corpus U papers analyzed with Seek & Blastn. Please note that some publications listed were incorrectly flagged by Seek & Blastn, and others contain no nucleotide sequence reagent errors

| **Corpus P PubMed ID’s** | **Corpus U PubMed ID’s** | | | |
| --- | --- | --- | --- | --- |
| 21287298 | 19732746 | 22072790 | 24694542 | 26893739 |
| 21573803 | 23383046 | 22155992 | 24814343 | 26919241 |
| 22901129 | 23869222 | 22156373 | 24885288 | 26998078 |
| 23911301 | 25010867 | 22185393 | 24959289 | 27274275 |
| 24427329 | 20846397 | 22293781 | 25052921 | 27313713 |
| 24448637 | 21595884 | 22458379 | 25136578 | 27346559 |
| 24714960 | 22864671 | 22615908 | 25176399 | 27446247 |
| 24775638 | 23049798 | 22650359 | 25215329 | 27446435 |
| 24842331 | 23284640 | 22662241 | 25232213 | 27485743 |
| 25123458 | 23490231 | 22710444 | 25294836 | 27485791 |
| 25169742 | 23806108 | 22761708 | 25317073 | 27573585 |
| 25215606 | 24475120 | 22791942 | 25323114 |  |
| 25223638 | 24879149 | 22965687 | 25370813 |  |
| 25262828 | 11549320 | 22984609 | 25370920 |  |
| 25279970 | 16581947 | 23049731 | 25381814 |  |
| 25301753 | 16987424 | 23050783 | 25387670 |  |
| 25322973 | 17873971 | 23071862 | 25400745 |  |
| 25432697 | 17897439 | 23104178 | 25400777 |  |
| 25471990 | 18299278 | 23125081 | 25431427 |  |
| 25496438 | 18577219 | 23236234 | 25434862 |  |
| 25524330 | 18698023 | 23352643 | 25529407 |  |
| 25629696 | 18798870 | 23372675 | 25571951 |  |
| 25643992 | 19107116 | 23538603 | 25624720 |  |
| 25676706 | 19123943 | 23578185 | 25625591 |  |
| 25703929 | 19201387 | 23613813 | 25669971 |  |
| 25737023 | 19266599 | 23638004 | 25799022 |  |
| 25746840 | 19564402 | 23638217 | 25825239 |  |
| 25833696 | 19755388 | 23685956 | 25889214 |  |
| 25889525 | 19893492 | 23687415 | 25954138 |  |
| 25893892 | 19920201 | 23705783 | 26045788 |  |
| 25932170 | 19942713 | 23974097 | 26137034 |  |
| 26008152 | 20023691 | 23983589 | 26137143 |  |
| 26056476 | 20394835 | 24040910 | 26157321 |  |
| 26303214 | 20531280 | 24073379 | 26171017 |  |
| 26448925 | 20661471 | 24092860 | 26300649 |  |
| 26472971 | 20805979 | 24133592 | 26309496 |  |
| 26550235 | 20934433 | 24172981 | 26310274 |  |
| 26581508 | 20946871 | 24228091 | 26339341 |  |
| 26601916 | 21283762 | 24272675 | 26339351 |  |
| 26823768 | 21311599 | 24348824 | 26395974 |  |
| 26844847 | 21327297 | 24370119 | 26440147 |  |
| 26902787 | 21375766 | 24387290 | 26544536 |  |
| 26937209 | 21385902 | 24604387 | 26569226 |  |
| 27044563 | 21537492 | 24618206 | 26575170 |  |
| 27130037 | 21869593 | 24650096 | 26622375 |  |
| 27174009 | 21923915 | 24651440 | 26722269 |  |
| 27177590 | 21987623 | 24658464 | 26856755 |  |
| 27313751 | 22004682 | 24660028 | 26870184 |  |
