# Supplementary material for: Semi-automated fact-checking of nucleotide sequence reagents in biomedical research publications: The Seek & Blastn tool
Source: PLoS One. 2019 Mar 1;14(3):e0213266. doi: 10.1371/journal.pone.0213266 (PMC6396917; doi:10.1371/journal.pone.0213266)
Supplement: S4 Table — (DOCX) [file pone.0213266.s004.docx]

S4 Table. Incorrectly identified nucleotide sequence reagents from Corpus P and/or Corpus U papers

| **Blasn verified identity** | **Nucleotide sequence reagents (5’→3’) detected by seek & blastn analysis** | **Published identity/ identities** |
| --- | --- | --- |
| *ACTB* | GTGGACATCCGCAAAGAC | *GAPDH* forward |
| *ACTB* | AAAGGGTGTAACGCAACTA | *GAPDH* reverse |
| *ACTB* | GGCGGCACCACCATGTACCCT | *GAPDH* forward |
| *ACTB* | AGGGGCCGGACTCGTCATACT | *GAPDH* reverse |
| *ANLN* | ATGCAGTGTGGTGCACATTT | *TPD52L2* forward |
| *ANLN* | AACCCAAACACTTTGGCAAG | *TPD52L2* reverse |
| *AXL* | GGAGGAAGCAATGATCT | *ACTIN* forward |
| *B2M* | ACCCCCACTGAAAAAGATGA | *ACTB* forward |
| *CTNNBIP1* | AGCTACTGCCTCCGGTCTTC | *CTNNB1* forward |
| *CTNNBIP1* | GTGGTCAACAGCCAGCTCA | *CTNNB1* reverse |
| *GABARAPL1/3* | ACTCCCACCCCACAAAATCC | *GABARAP* forward |
| *GAPDH* | **CCAGCCCCAGCGTCAAAGGTG**^a^ | *GLI1* reverse |
| *GYPC* | CTAGGTGTTCTAGTCTGGACT | Non-targeting shRNA |
| *HDGF* | GAGGGTGACGGTGATAAGAA | *HOXA1/ADAM8* forward |
| *HDGF* | GAAACATTGGTGGCTACAGG | *HOXA1/ADAM8* reverse |
| *HDGF* | CAGCCAACAAATACCAAGTCT | *HOXA1/ADAM8* forward |
| *HDGF* | GTTCTCGATCTCCCACAGC | *HOXA1/ADAM8* reverse |
| *HDGF* | CACCGCCGTGAAATCAACAGCCAAAACGTTGGCTGTTGATTTCACGG | *HOXA1/ADAM8* shRNA sense |
| *HDGF* | AAAACCGTGAAATCAACAGCCAACTTTTGGCTGTTGATTTCACGGC | *HOXA1/ADAM8* shRNA antisense |
| *HDGF* | CACCGCCCTGAATTGAACAGCCAAAACGTTGGCTGTTGATTTCACGG | Non-targeting shRNA sense |
| *NOB1* | CTAGCCCGGCCAAGGAAGTGCAATTGGTTTTTTGTTAAT | Non-targeting shRNA |
| *NOB1* | CTAGCCCGGCCAAGGAAGTGCAATTGCATACTCGAGTATGCAATTGCACTTCCTTGGTTTTTTGTTAAT | Non-targeting shRNA |
| *NOB1* | CTAGCCCGGCCAAGGAAGTGCAATTGCATACTCGAGTATGCAATTGCACTTCCTTGGTTTTTGTTAAT | Non-targeting shRNA |
| Non-targeting^b^  (Rice field eel) | TGGGTGTGATGGTTGGCATGG | *ACTB* forward |
| Non-targeting  (Rice field eel) | TAAGAAAGATGGCTGGAAGAGGG | *ACTB* reverse |
| Non-targeting | ATCTTCAAACCTCATGAT | *ACTB* reverse |
| Non-targeting | GGAGTCTCACCCACCACCCT | *BAX* reverse |
| Non-targeting | AUUUACUACUAAAUCCUCCCCC | *CHRM2* siRNA sense |
| Non-targeting | GGGGGAGGAUUUAGUAGUAAAU | *CHRM2* siRNA antisense |
| Non-targeting | GGGGGAAGAAAUGGGCUACUA | *CHRM2* siRNA antisense |
| Non-targeting | GGGGGUUAGGUCAAGGUGUAACU | *CHRM2* siRNA antisense |
| Non-targeting | GGTCGACCTAGGCATCAAGATTGCTATC | *CIAPIN1* reverse |
| Non-targeting | ATATGGATCCATGCAAAATCGAGCTCGAGA | *CTDP1* forward |
| Non-targeting | GCGGCCGCTAATCTTCAATTTACCCTAATA | *CTDP1* reverse |
| Non-targeting | CCCTCCCTTGGTTATGATCCA | *GABARAPL1* forward |
| Non-targeting | AGGAAGGGATTGCTGGGTTCT | *GABARAPL1* reverse |
| Non-targeting | GCCTCAAGATCAGCAAT | *GAPDH* forward |
| Non-targeting | GTTCCAGTATGACTCTACCC | *GAPDH* forward |
| Non-targeting | AGTCTTCTGAGGCAGTGATG | *GAPDH* reverse |
| Non-targeting | GGTATCGGAAGGACTC | *GAPDH* forward |
| Non-targeting | UCAUGUUUCUCUCUACGAdTdT | *HOXA13* siRNA sense |
| Non-targeting | GGAATCGTGGTCTCTCACTCG | *LIN28* forward |
| Non-targeting | GTGTGATGTCCGGACTGTCAT | *LIN28* reverse |
| Non-targeting | CTGTAACGTTGCGAATGGTAT | *LIN28* shRNA target sequence |
| Non-targeting  (rat) | CCGCCTCAGTATGCAGTCCA | *MAPK* forward |
| Non-targeting | AATTCAAAAAATAGCTTATCAG | *MIR21* reverse |
| Non-targeting | CTAGCCCGGTTCTCCGAACGTGTCACGTATCTCGAGATACGTGACACGTTCGGAGAATTTTTTTAAT | *NOB1* shRNA |
| Non-targeting | CTCTAGACATGATCTCTTTTCACACAGC | *NOB1* miR-646 mutagenesis primer |
| Non-targeting  (mouse) | ACGGAGTACGGCCCATGTT | *NOTCH1* reverse |
| Non-targeting  (mouse) | GCTGACATCGGACACTTA | *PCNA* forward |
| Non-targeting  (mouse) | CTCAGGTACAAACTTGGTG | *PCNA* reverse |
| Non-targeting | CCGGAATTCTCAGACGTTACTTTCACCGTGCC | *SKP2* reverse |
| Non-targeting | ATGTGATTTACTGCTGCGTGT | *SMAD8* forward |
| Non-targeting | AACGGTACTCCGCCACC | *TP53* forward |
| Non-targeting  (rat) | CTGACTCTCGGACCCCTG | *VEGFR-1* forward |
| Non-targeting | GCACCACCAACGGAATCG | RNA18S reverse |
| *PNO1* | CCGGGCTGAACAATTTCAGTCATTTCTCGAGAAATGACTGAAATTGTTCAGCTTTTTG | *NOB1* shRNA |
| *TPD52L2* | TTCACAGGCAGGACAGAAGA | *ACTIN* forward |
| *TPD52L2* | TTGAAGGTCGCAGAGTTCCT | *ACTIN* reverse |
| *TPD52L2* | GCGGAGGGTTTGAAAGAATATCTCGAGATATTCTTTCAAACCCTCCGCTTTTTT | Non-targeting shRNA |

| **Blastn verified identity** | **Nucleotide sequence reagents (5’→3’) detected by manual analysis** | **Published identity** |
| --- | --- | --- |
| *ACTB* | AAAGACCTGTACGCCAACAC | U6 forward primer |
| *BIRC3* | GCATACTGAGACCCTG | *BCL2* forward |
| *CEP104* | GAAGCAGCAGAUGGAGCTT | *STAT3* siRNA sense |
| *CEP104* | GCUCCAUCUGCUGCUUCTT | *STAT3* siRNA antisense |
| *EZH2* | AGCAACUGCAUUCAGAGUCTT | Control siRNA |
| *GAPDH* | **CCAGCCCCAGCGTCAAAGGTG**^a^ | *ACTB* reverse |
| *GAPDH* | CGCGGGCTCTCCAGAACATCAT | *ACTB* forward |
| *GLP2R* | TGGTGCATGGTCCTGTTG | *VEGFR-1* reverse |
| *GPR137* | ACCTGGGGAACAAAGGCTAC | *ZFP91-P* forward |
| *GPR137* | TAGGACCGAGAGGCAAAGAC | *ZFP91-P* reverse |
| *HDGF* | AAAACCGTGAAATCAACAGCCAACTTTTGGCTGTTCAATTCAGGGC | Non-targeting shRNA antisense |
| *H19* | CTGTCCTCGCCGTCACACCG | *GAPDH* forward |
| *H3F3A* | GCTAGCTGGATGTCTTTTTGG | H3B2 forward |
| *H3F3A* | GTGGTAAAGCACCCAGGAAA | H3B2 reverse |
| *KLF8* | GGAGTGTTGGAGAAGTCATATTAC | *GAPDH* reverse |
| *KLK14* | ACGCACCCCAACTACAACTC | ACTB reverse |
| *KIAA0125* | CCTCTCAGCCTCCAGCGTTG | linc-*ITGB1* forward |
| *KIAA0125* | TGCTCTTGCTCACTCACACTCC | linc-*ITGB1* reverse |
| Non-targeting | GCAACAGTTGCAGAGAGGU | *ACTB* siRNA antisense |
| Non-targeting  (mouse) | AAGTAGAAGAGGGCAACCAC | *BAX* reverse |
| Non-targeting | CACTCAGAGGAGGAAAATCCAGT | *CDKN1A* (P21) forward |
| Non-targeting | AUGUAGCCCAUUUCUUCCCCC | *CHRM2* siRNA sense |
| Non-targeting | UCCUUUGAGUUUCAGGCUGCCCCC | *CHRM2* siRNA sense |
| Non-targeting | GGGGGCAGCCUGAAACUCAAAGGA | *CHRM2* siRNA antisense |
| Non-targeting | AGUUACACCUUGACCUAACCCCC | *CHRM2* siRNA sense |
| Non-targeting | CATGUGUTGGUAAGCUCUA | *CTNNB1* siRNA |
| Non-targeting  (rat) | GCCACATGTGCAAAGGCATC | *MAPK* reverse |
| Non-targeting | AGCTTGGCGTAATCATGGTCATAGCTGTTTCCTGTGTGAAATTGTTATCCGCTCACAATTCCACACA | *MAWBP* shRNA reverse |
| Non-targeting | CGGGTTATTGCTGACAGACGC | *RNA45S5* |
| *SMAD9* | CGGTAGTGGTAAGGGTTAATGC  PrimerBank: GGCGGTAGTGGTAAGGGTTAAT^c^ | *SMAD8* reverse |
| *TP53* | CCTCCTCAGCATCTTATCCG | *BIRC5* forward |
| *TP53* | CACAAACACCCACCTCAAA | *BIRC5* reverse |
| *TUB* | GAAGGCCTCATCCTCCACTTTGGAAAG | *TUBB* forward |
| *TUB* | TGCTAGCAGTGTCTCATGCTCG | *TUBB* reverse |
| *TUB* | GCATCAGTAGCTGAGTGCACTCCTGGT | *TUBB* forward |
| *TUB* | GTAGAGGGTATGAAGGGCAAGAACTCT | *TUBB* reverse |

^a^Identical nucleotide sequences shown in bold were identified using Seek & Blastn and manual analysis in different papers.

^b^Species information is provided for non-targeting sequences employed in experiments in non-human systems. All other non-targeting sequences were used to analyse human systems.

^c^Nucleotide sequence is aligned with *SMAD9* primer sequence from PrimerBank (ref. 47).
